# Prediction of Aging Performance of Particle-Filled Polymer Composites Based on RVE Model

**DOI:** 10.3390/polym15244724

**Published:** 2023-12-16

**Authors:** Huizhen Wang, Congli Fang, Yujiao Zhang, Minghua Zhang, Tao Shen, Jianke Du

**Affiliations:** Smart Materials and Advanced Structure Laboratory, School of Mechanical Engineering and Mechanics, Ningbo University, Ningbo 315211, China; wwwhz777@163.com (H.W.); 2011081025@nbu.edu.cn (C.F.); 2111081032@nbu.edu.cn (Y.Z.); zhangminghua@nbu.edu.cn (M.Z.)

**Keywords:** particle-filled polymer composite, thermo-oxidative, representative volume element, cohesive zone model

## Abstract

In this study, the aging performance of particle-filled polymer composites (PFPCs) under thermo-oxidative conditions was investigated on multiple scales. High-temperature-accelerated tests were conducted to analyze the effects of aging time and temperature. A representative volume element (RVE) model was established for the PFPCs using a random particle-filling algorithm. A predictive model for the crosslink density was conducted based on the closed-loop chain reaction of polymer oxidation. According to the theory of polymer physics, the relation between the crosslink density and matrix modulus was determined. The particle/matrix interface in the RVE model was represented by the cohesive zone model (CZM). The parameters of the CZM were determined by the inversion techniques. Then, a comprehensive multiscale RVE model was constructed, which was applied to predict the modulus and dewetting strain of the aged PFPCs. The predicted results show good agreement with the test results, which verifies the reliability of our model.

## 1. Introduction

Particle-filled polymer composites (PFPCs), as a typical type of advanced composite material, have been widely applied in industries such as machinery, aerospace, automotive manufacturing, and medical devices [1,2,3]. In PFPC systems, it is common to select a high-molecular-weight polymer with good flexibility as the matrix and fill it with high-rigidity, high-strength inorganic particles. These materials not only retain excellent properties of polymers, such as high specific strength, corrosion resistance, and good elasticity, but also exhibit good dimensional stability and creep resistance, showing outstanding comprehensive mechanical performance. PFPCs offer vast potential and a wide range of applications. However, the performance of polymer matrices may degrade significantly due to their long-term exposure to extreme environments [4,5]. High-temperature oxidation or thermo-oxidative aging is one such example. A series of chemical reactions occur in the polymer matrix, driven by oxygen (O_2_) diffusion, which may cause microcracking in the matrix, debonding on the particle–matrix interface, and, eventually, the failure of the material. Therefore, thermo-oxidative aging is one of the major concerns in the long-term use of PFPCs.

Thermo-oxidative aging in polymers and PFPCs involves a multiscale phenomenon that starts at a molecular level with a set of irreversible chemical reactions, leading to changes in the microscale molecular configuration and macroscale mechanical properties. As oxygen infuses into the polymer, chain scission and oxidative crosslinking occur, which cause an increase in the modulus and a decrease in failure strain [6,7,8,9]. In addition, the deterioration of the interface strength in PFPCs has also been reported [10]. The prediction of thermo-oxidative aging performance is a challenging task. Traditionally, Arrhenius law [11,12] is often used to extrapolate the service life for long-term operating temperatures based on accelerated aging tests. However, it is well known that Arrhenius’s law can be applied only to an elementary reactive process, while thermo-oxidative aging of polymers includes at least six elementary reactions [13]. A generalized Arrhenius law might not accurately describe the aging process.

Currently, the closed-loop chain reaction for thermo-oxidative aging [14] has been extensively studied, and a kinetic model was formed to track the aging process. A predictive model for the aging effect on modulus and fracture toughness of polymers based on the closed-loop chain reaction has been reported in several pieces of literature [15,16]. In the case of PFPCs, the aging performance of the particle/matrix interface is crucial for predicting the macroscopic aging characteristics. However, due to the difficulty in measuring the interface strength, quantitative research on the degradation of interface strength during aging is limited. Representative volume element (RVE)-based [17,18,19] mesoscale and microscale models play important roles in studying the multi-scale mechanical properties of PFPCs. In particular, the RVE combined with the cohesive zone model (CZM) [20,21,22] has been widely used to simulate particle debonding in PFPCs, which is also named dewetting in some of the literature [23,24,25].

Thus, in this study, an RVE-based mesoscale model for the PFPCs was constructed. Then, a predictive model for the polymer matrix modulus was developed based on the closed-loop chain reaction of thermo-oxidative aging. After that, the parameters of the CZM introduced into the RVE model were determined based on an inverse analysis approach. Eventually, a novel RVE model for predicting the multiscale aging performance of PFPCs was developed.

## 2. Materials and Methods

**Figure 1:**
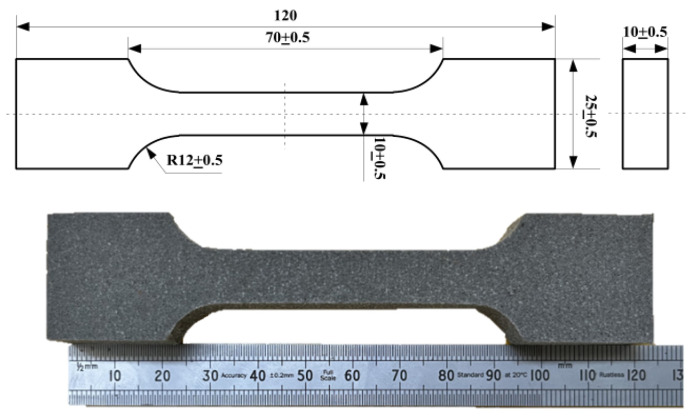
Diagram of specimen (Unit: mm).

In this study, we collaborated with the manufacturer to obtain a batch of PFPC material specimens. The matrix materials consist of hydroxyl-terminated polybutadiene and toluene diisocyanate, with fillers comprising ammonium perchlorate particles. Additionally, a small quantity of aluminum (Al) was added to enhance the energy density. The styles and sizes of PFPC specimens are shown in Figure 1. To assess the impact of aging on the specimens, we conducted high-temperature-accelerated aging tests. The PFPC specimens were sealed in aluminum foil pouches and then placed in a curing constant temperature test chamber, model LC-101-0B (Shanghai Lichen Instrument Technology Co., LTD, Shanghai, China), to conduct the accelerated aging experiments under the conditions listed in Table 1 [26].

**Figure 2:**
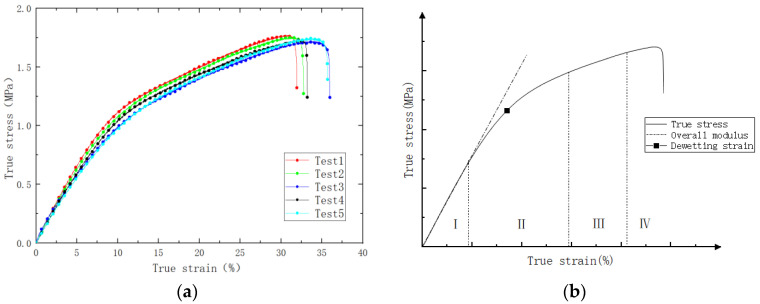
(**a**) Experimental true stress-true strain curve; (**b**) definition of dewetting strain.

**Figure 3:**
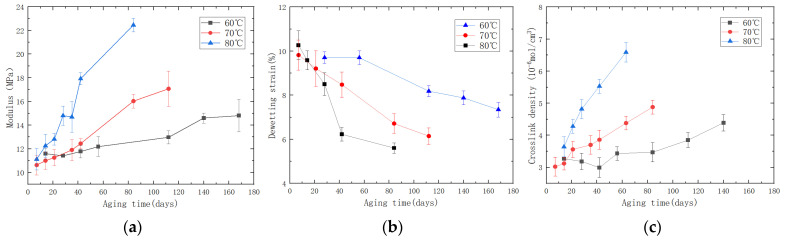
(**a**) Line chart of the relationship between the modulus and aging time; (**b**) line chart of the relationship between dewetting strain and aging time; (**c**) line chart of the relationship between crosslink density and aging time during the accelerated aging process.

After the specified aging time, the PFPC specimens were removed and left in a desiccator for natural cooling for one day to allow stress relief. Subsequently, uniaxial tensile tests were conducted using the AGS-X (SHIMASZU, Shanghai, China) universal testing machine to evaluate various mechanical properties of the PFPCs. The tensile tests were performed at room temperature with a testing temperature of 25 ± 2 °C and a relative humidity not exceeding 70%. The universal testing machine was set to a constant rate of extension mode with a stretching speed of 100 mm/min, continuing until complete fracture and separation of the test specimen occurred. The stress–strain curves obtained are shown in Figure 2a. It can be seen from Figure 2b that the stress–strain curve can be divided into four stages: In the initial stage, the material exhibits good linear elasticity. With the increase in strain, nonlinearity appears due to the debonding of the particles and matrix. The point at which the slope experiences the maximum change is defined as a dewetting point [27,28,29]. The third stage is the matrix-bearing stage, during which the interface fails and the matrix bears the load. The fourth stage is the damage stage of the matrix, which leads to the final fracture of the PFPC material. In this study, the aging performance of the PFPCs in stages I and II was investigated, and the damage to the matrix was neglected. Stress–strain curves were processed to obtain the modulus and dewetting strain of aged PFPCs, as shown in Figure 3a,b. With the increase in aging time, the modulus of PFPCs increased continually while the dewetting strain decreased.

**Figure 4:**
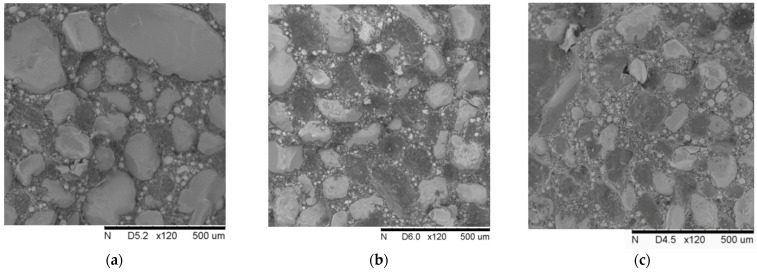
SEM surface topography of tensile fracture section for (**a**) unaged, (**b**) aged at 80 °C for 42 days, and (**c**) aged at 80 °C for 84 days.

With the aging of PFPCs, chain scission and chain crosslinking occur constantly, and the chemical structure of the polymer matrix changes. Crosslink density is an important index to characterize the degree of crosslinking of polymers. In this study, a balanced swelling method was used to measure the crosslink density of aging specimens [30]. The obtained results are shown in Figure 3c. As the aging time increases, there is a noticeable increase in crosslink density. To visually observe the impact of aging time on particle dewetting, we extracted block-shaped specimens measuring 10 mm × 10 mm × 4 mm from the fracture and side surfaces of the PFPC specimens after the tensile tests. These samples underwent vacuum drying and conductive coating treatment. Surface morphology observations were conducted using a TM3000 (Hitachi, Tokyo, Japan) scanning electron microscope. The SEM images of the tensile fracture surface of the PFPC specimens are shown in Figure 4. From left to right, the samples shown are unaged, aged at 80 °C for 42 days, and aged at 80 °C for 84 days. It can be obviously found that the volume fraction of particles in the PFPC specimens is high, which can reach about 70%. With the increase in aging time, the particles are more easily pulled off under a tension load, and more holes are left by interface debonding. This phenomenon indicates that with the increase in aging time, the strength of the particle/matrix interface decreases.

## 3. Multiscale Aging Model

### 3.1. RVE Model of PFPCs

**Figure 5:**
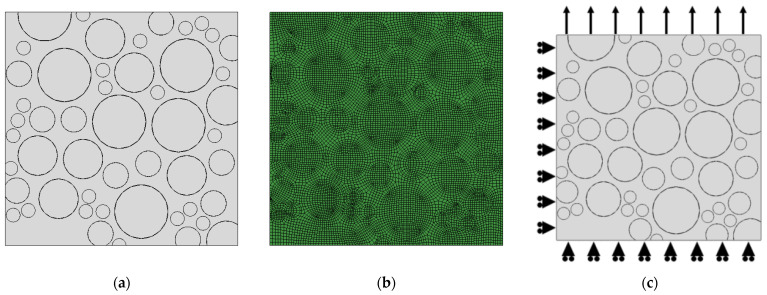
(**a**) Random particle-filling model; (**b**) quadrilateral mesh division with a size of 0.02 mm; (**c**) uniform displacement boundary condition.

Through observations of the surface topography of PFPCs, it was observed that the surface of Al is smooth, and it adheres well to the matrix. The debonding of Al from the matrix is rarely observed. Therefore, only the matrix and AP particles are considered in our model. According to the SEM surface topography, the volume fraction of the particles is about 70%. Following the molecular dynamics method proposed by Knott [31], we randomly distributed a certain number of points (zero-radius particles) within the computational domain to establish the initial configuration and assigned random velocities and radius growth rates to the particles. The particles continued to grow and collide within the computational domain until the volume fraction reached 70%. The FEA model was constructed by introducing the random particle-filling model into ABAQUS with the Python script, as shown in Figure 5a. We selected a quadrilateral mesh with a grid size of 0.02 mm, and the mesh partitioning result is shown in Figure 5b. To simulate the formation and propagation of interface debonding between the particles and matrix, we introduced a cohesive layer. In total, the model consists of 13,043 nodes and 12,878 elements, including 4038 CPE4H elements for the matrix, 7270 CPE4 elements for the particles, and 1570 cohesive elements for the interface. Uniform displacement boundary conditions were applied, as shown in Figure 5c.

**Figure 6:**
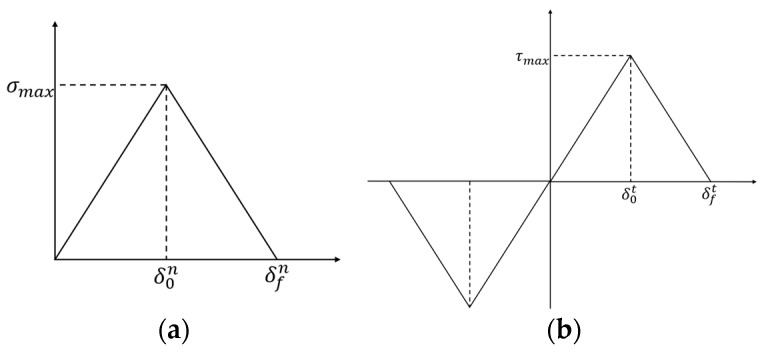
(**a**) Constitutive relationship of bilinear CZM between normal stress and normal strain; (**b**) constitutive relationship of bilinear CZM between shear stress and shear strain.

The constitutive relationship of the CZM is shown in Figure 6, which can be illustrated with Equations (1) and (2):

(1) $$\sigma =\left\{\begin{array}{cc} K_{1}^{n}\delta & 0\leq \delta \leq \delta_{0}^{n}\\ \left(1+{K_{2}^{n}/K}_{1}^{n}\right)\sigma_{max}-K_{2}^{n}\delta & \delta_{0}^{n}\leq \delta \leq \delta_{f}^{n}\\ 0 & \delta_{f}^{n}>\delta \end{array}\right.,$$

(2) $$\tau =\left\{\begin{array}{cc} K_{1}^{t}\delta & 0\leq \delta \leq \delta_{0}^{t}\\ \left(1+{K_{2}^{t}/K}_{1}^{t}\right)\tau_{max}-K_{2}^{t}\delta & \delta_{0}^{t}\leq \delta \leq \delta_{f}^{t}\\ 0 & \delta_{f}^{t}>\delta \end{array}\right.,$$

where σ is the normal stress; τ is the shear stress; and $\sigma_{max}$ and $\tau_{max}$ represent the maximum stress values in normal and tangential directions (i.e., interface strength), respectively. $\delta_{0}^{n}$ and $\delta_{0}^{t}$ represent the normal and tangential interfacial opening displacements, respectively. $\delta_{f}^{n}$ and $\delta_{f}^{t}$ represent the normal and tangential interfacial failure displacements, respectively. $K_{1}^{n}$ and $K_{1}^{t}$ represent the normal and tangential stiffness of a cohesive element, respectively. $K_{2}^{n}$ and $K_{2}^{t}$ represent the normal and tangential softening modulus, respectively. To simplify the simulation, $\sigma_{max}=\tau_{max}$, $\delta_{0}^{n}=\delta_{0}^{t}$, $\delta_{f}^{n}=\delta_{f}^{t}$, $K_{1}^{n}=K_{1}^{t}$, $K_{2}^{n}=K_{2}^{t}$ were assumed. In the constitutive relation of the CZM, the initial stiffness of the interface serves as a non-physical quantity that ensures a rigid connection between the upper and lower interfaces before any damage occurs [32]. Thus, a high stiffness of 1200 MPa/mm was applied by considering the computational convergence. In addition, the aging effect on failure displacement was neglected, which was chosen as 0.02 mm.

**Figure 7:**
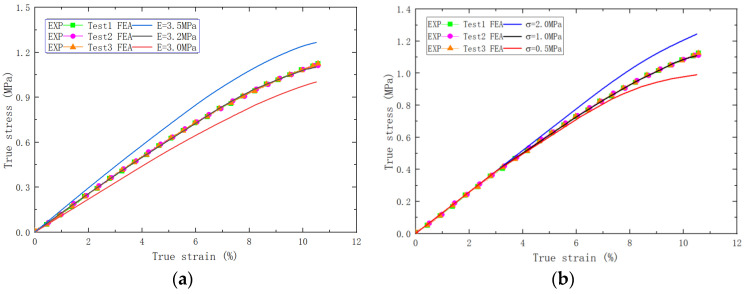
(**a**) The comparative diagram of experimental/simulation stress–strain curves with different matrix moduli. (**b**) The comparative diagram of experimental/simulation stress–strain curves with different interface strengths.

The elastic modulus of the particles was chosen as 32.5 GPa [33], and the effect of aging on the modulus of the particles was disregarded. The modulus of the matrix and the strength of the CZM were determined by fitting the FEA results to the test results, as shown in Figure 7. It can be seen that the influence of the matrix modulus is mainly reflected in the elastic stage of the results, while the influence of the interface strength is mainly reflected in the interface debonding stage. The modulus of matrix ($E_{0}$) and the strength of the interface were determined as 2.3 MPa and 1.11 MPa, respectively, for the unaged PFPC specimens.

### 3.2. Matrix Modulus Prediction Based on Chain Reaction

#### 3.2.1. Chemistry of Polymer Oxidation

The Arrhenius equation is commonly employed to describe the aging of a polymer to determine its lifetime. This equation essentially represents the relationship between the rate constant k of an elementary process and the absolute temperature T, as shown in Equation (3):

(3) $$k=A\;exp\;\left[-E_{a}/\left(R\;T\right)\right],$$

where *A* (pre-exponential factor) and *E_a_* (activation energy) are characteristic of this process, and *R* is the gas constant.

Actually, the aging of the polymer matrix is a complex chemical reaction consisting of several fundamental reactions at the molecular scale. Research [14] has listed the simplest oxidative aging model with six sets of closed-loop chain reactions involving three major steps, identified as initiation, propagation, and termination, as shown in Table 2.

In the reaction stage of propagation, the rate constant k_II_ for reaction (II) is much faster than the rate constant k_III_ for reaction (III). Therefore, in the case of excess availability of oxygen, reaction (III) is the main factor limiting the reaction rate. At this point, due to the presence of excess oxygen, alkyl radical (P*) is rapidly transformed into peroxy radical (PO_2_*). The termination reaction occurs mainly through reaction (VI). Thus, the constant rate of oxygen consumption k_1_ can be obtained as $k_{1}=k_{III}\;\sqrt{k_{I}\;/\;2k_{VI}}$ based on the reaction equilibrium. On the other hand, in the case of limited oxygen availability, reaction (II) is the main factor limiting the reaction rate. At this point, limited oxygen leads to the accumulation of a large number of alkyl radicals (P*), which cannot be converted into peroxide radicals (PO_2_*). The termination reaction occurs mainly through reaction (IV). The constant oxygen consumption rate k_2_ can be obtained as $k_{2}=k_{II}\;\sqrt{k_{I}\;/\;2k_{IV}}$ based on the reaction equilibrium. To calculate the oxygen consumption rate under different oxygen concentrations, the hyperbolic model was selected [34], which can represent the oxygen consumption rate according to the concentration of oxygen and polymer substrate:

(4) $$S_{Ox}=\frac{k_{1}{\;k}_{2}\;\left[O_{2}\right]\left[PH\right]}{k_{2}\;\left[O_{2}\right]+k_{1}\;\left[PH\right]},$$

where [O_2_] represents the concentration of oxygen in the specimen, and [PH] represents the concentration of the polymer matrix in the specimen.

#### 3.2.2. Multiscale Predictive Model of Matrix Modulus

Since the specimens used in the accelerated aging test are relatively thin, the oxygen dissolved on the surface can easily penetrate and diffuse into the interior. Therefore, the oxygen concentration inside ([$O_{2}$]) can be approximated by the concentration of oxygen on the surface (${\left[O_{2}\right]}_{s}$), as shown in Equation (5):

(5) $$\left[O_{2}\right]\approx {\left[O_{2}\right]}_{s}=Sol_{O_{2}}\cdot p_{O_{2}}={Sol}_{O_{2}0}\cdot exp\left[{-H}_{S_{O_{2}}}/\left(R\;T\right)\right]\cdot p_{O_{2}},$$

where ${Sol}_{O_{2}0}$ is the basic coefficient of oxygen solubility; $H_{S_{O_{2}}}$ is the enthalpy of $O_{2}$ in the matrix; and $p_{O_{2}}$ is the partial pressure of oxygen in the atmosphere. The concentration of the matrix can be expressed as Equation (6):

(6) $$\left[PH\right]=\left(\;\rho_{PH}/M_{PH}\;\right)\;\left(\;V_{PH}/V\;\right),$$

where $\rho_{PH}$ is the density of the matrix; $M_{PH}$ is the molar mass of the matrix; $V_{PH}$ is the volume of the matrix; and V is the total volume of the specimen.

In calculating the crosslink density, we exclusively accounted for the changes resulting from the reaction with oxygen. The alteration in crosslink density is directly proportional to the amount of oxygen consumed due to the oxidation of the matrix. Therefore, the local crosslink density of the matrix can be estimated as Equation (7):

(7) $$\nu =\nu_{0}+\omega \;S_{Ox}\;t,$$

According to classical theory, the shear modulus of an ideally crosslinked polymer is directly proportional to the crosslink density. For our PFPC materials, with a matrix Poisson’s ratio of 0.495, their elastic modulus is also directly proportional to the crosslink density, as shown in Equation (8):

(8) $$E=E_{0}\;\nu /\nu_{0},$$

where $E_{0}$ is the matrix modulus without aging, which is obtained by simulation inversion, $E_{0}$ = 2.3 MPa.

Therefore, the prediction model of the matrix modulus is obtained, as shown in Equation (9):

(9) $$E=2.3\times \left(\;1+\frac{\omega {\;A}_{1}{\;e}^{\frac{-E_{a1}}{R\;T}}{\;Sol}_{O_{2}0}\;e^{\frac{-H_{sO_{2}}}{R\;T}}\;p_{O_{2}}\;t}{\nu_{0}\;\left(\;1+\frac{A_{1}{\;e}^{\frac{-E_{a1}}{R\;T}}{\;Sol}_{O_{2}0}{\;e}^{\frac{-H_{sO_{2}}}{R\;T}}\;p_{O_{2}}}{A_{2}{\;e}^{\frac{-E_{a2}}{R\;T}}\left(\;\rho_{PH}/M_{PH}\;\right)\;\left(\;V_{PH}/V\;\right)}\;\right)}\;\right),$$

#### 3.2.3. Parameters for Calculation of Matrix Modulus

In the multiscale predictive model of the matrix modulus, a total of 13 parameters need to be determined. Among them, the parameter values of R, $p_{O_{2}}$, ${Sol}_{O_{2}0}$, $H_{S_{O_{2}}}$, $E_{a1}$, and $E_{a2}$ can be found by referring to the relevant standards and literature [35,36]. The crosslink density values measured by the equilibrium swelling test were substituted into Equation (7) for fitting, and the parameter values of ω, $A_{1},$ and $A_{2}$ can be obtained. The fitting results of the parameters are shown in Table 3.

**Figure 8:**
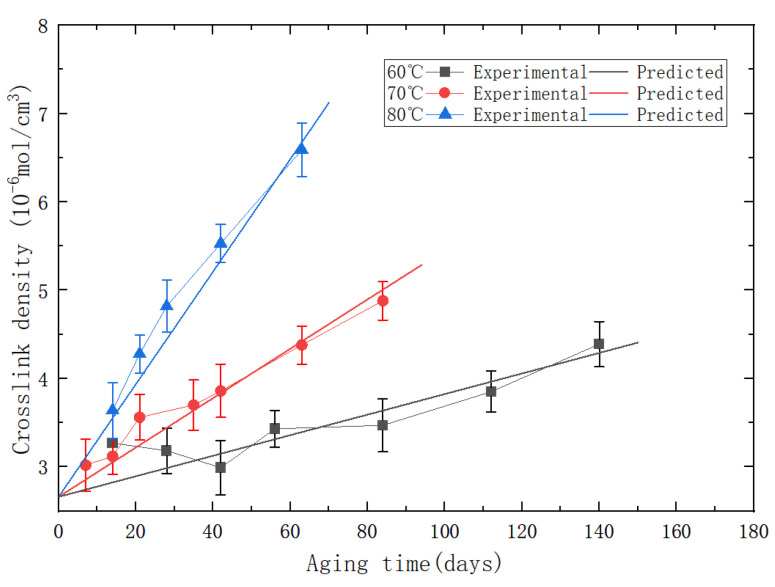
The comparative diagram of experimental/predicted crosslink density.

By substituting the parameters in Table 3 into Equation (7), a predictive model for the crosslink density of PFPC materials was obtained. The comparative diagram of experimental/predicted crosslink density is shown in Figure 8.

According to calculations, the $R^{2}$ value of the predictive model for the crosslink density is 0.91, which indicates that the predictive model has an acceptable accuracy and reliability. By substituting the parameters in Table 3 into Equation (9), the predictive model for the matrix modulus of the aged PFPCs can be obtained, as shown in Equation (10):

(10) $$E=2.3+\frac{2.4\times 10^{14}\times e^{\frac{-6527}{T}}\times t}{1+1.8\times {10^{-3}\times e}^{\frac{3532}{T}}}$$

### 3.3. Interface Strength Prediction Based on Inversion Analysis

#### 3.3.1. Inversion of Interface Strength

**Figure 9:**
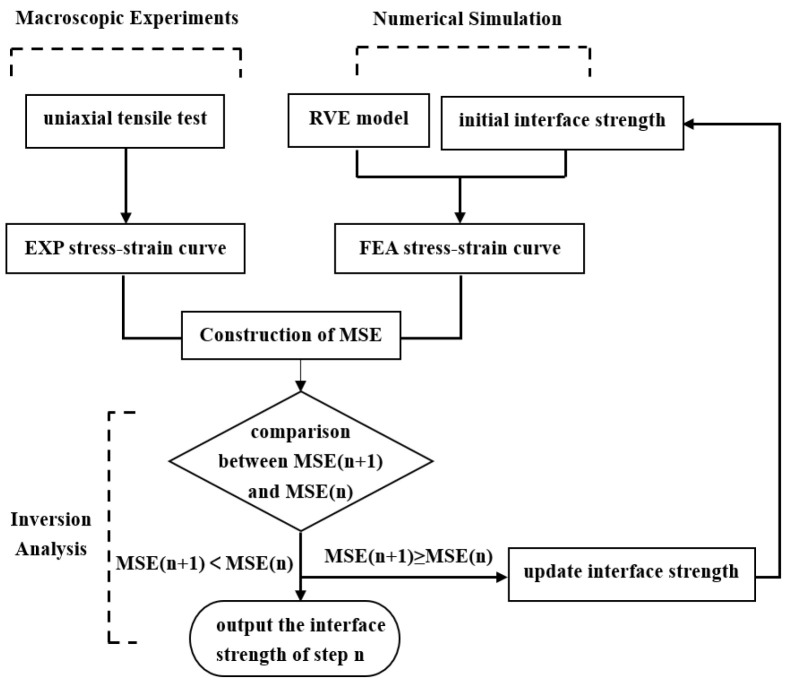
Inversion process of interface strength.

The parameter inversion analysis method is applied to determine the strength of the interface for the aged PFPCs. By continuously adjusting the simulation parameters and optimizing the computational results, an accurate set of model parameters can be obtained, as illustrated in Figure 9. The mean square error (MSE) is defined as Equation (11).

(11) $$MSE=\frac{1}{5}\;\sum_{i=1}^{5}\;{\left({\;\sigma }_{i}^{FEA}-{\;\sigma }_{i}^{EXP}\;\right)}^{2}$$

**Figure 10:**
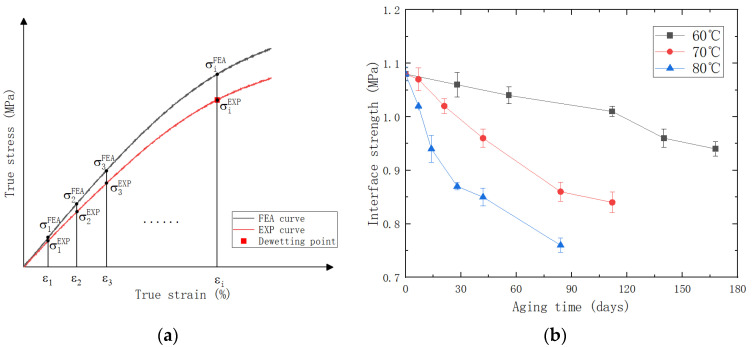
(**a**) Establishment of objective function MSE; (**b**) relationship between interface strength and aging time.

Five points at equal intervals under the dewetting strain were taken as reference data, as shown in Figure 10a. The initial interface strength is chosen based on the stress values corresponding to the dewetting strain obtained from the experimental stress–strain curve. Then, we adjusted the interface strength value with an increment of 0.01 to achieve the minimum value of the MSE. The interface strength under different aging temperatures and aging times was obtained through the simulation. With the increase in aging time and aging temperature, an obvious decrease in interface strength can be observed, as shown in Figure 10b.

#### 3.3.2. Multiscale Predictive Model of Interface Strength


(12) $$\sigma =\alpha \;v^{\beta }$$

**Figure 11:**
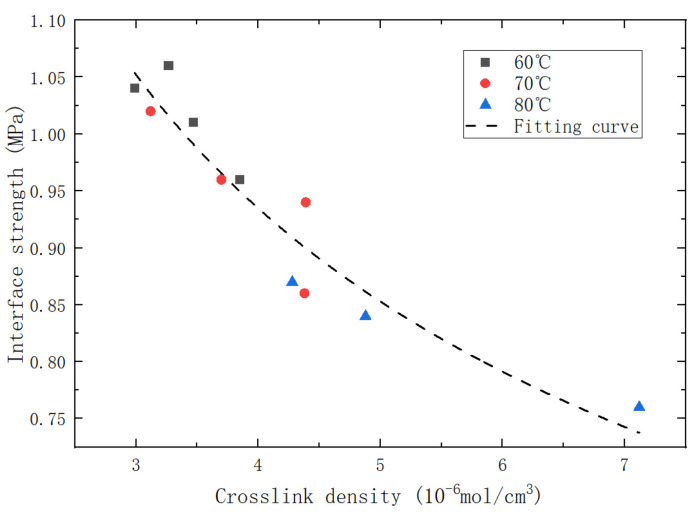
Diagram of crosslink density/interface strength fitting for the specimen.

To construct a unified multiscale model, we correlated the interface strength with the crosslink density of the matrix and fitted their relationship using a power function, as displayed in Figure 11. 

Then, the predictive model of interface strength is obtained, as shown in Equation (13):

(13) $$\sigma =\alpha \times {\left({\;\nu }_{0}+\frac{{\omega \;A}_{1\;}e^{\frac{-E_{a1}}{R\;T}}\;{Sol}_{O_{2}0}\;e^{\frac{-H_{sO_{2}}}{R\;T}}\;p_{O_{2}}\;t}{1+\frac{A_{1}{\;e}^{\frac{-E_{a1}}{R\;T}}\;{Sol}_{O_{2}0\;}e^{\frac{-H_{sO_{2}}}{R\;T}}\;p_{O_{2}}}{A_{2\;}e^{\frac{-E_{a2}}{R\;T}}\;\left(\;\rho_{PH}/M_{PH}\;\right)\;\left(\;V_{PH}/V\;\right)}}\;\right)}^{\beta }$$

**Figure 12:**
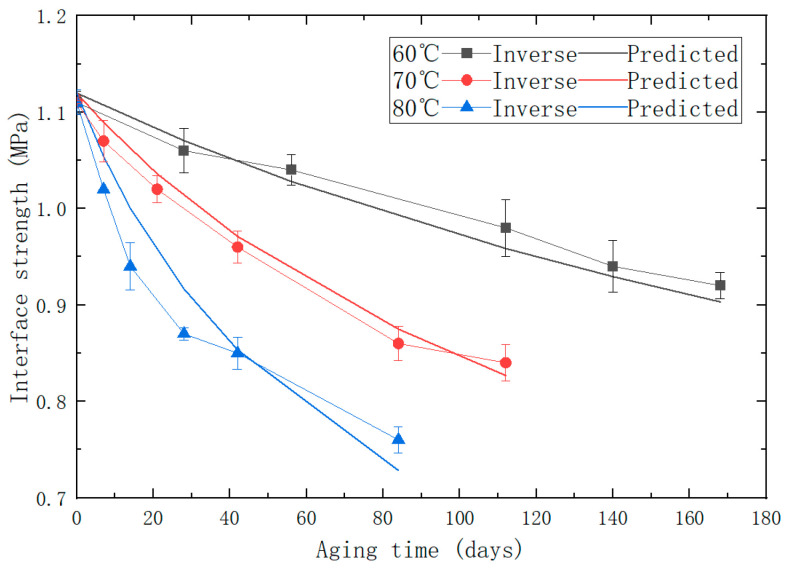
The comparative diagram of experimental/predicted interface strength.

The values of α and β can be obtained from the fitting results of the power function, which are: α=1.64, β=−0.39, and the remaining values can be referred to Table 3. The comparative diagram of the inverse/predicted interface strength is shown in Figure 12. According to calculations, the $R^{2}$ value of the predictive model for interface strength is 0.90, which indicates that the predictive model has an acceptable accuracy and reliability. By substituting the parameters into Equation (13), a multiscale predictive model for the interface strength of aged PFPCs is obtained:

(14) $$\sigma =1.64\times {\left(\;2.66+\frac{2.8\times 10^{8\;}\times {\;e}^{\frac{-6527}{T}}\times t}{1+1.8\times {{\;10}^{-3}\times e}^{\frac{3532}{T}}}\;\right)}^{-0.39}$$

## 4. Results and Discussion

**Figure 13:**
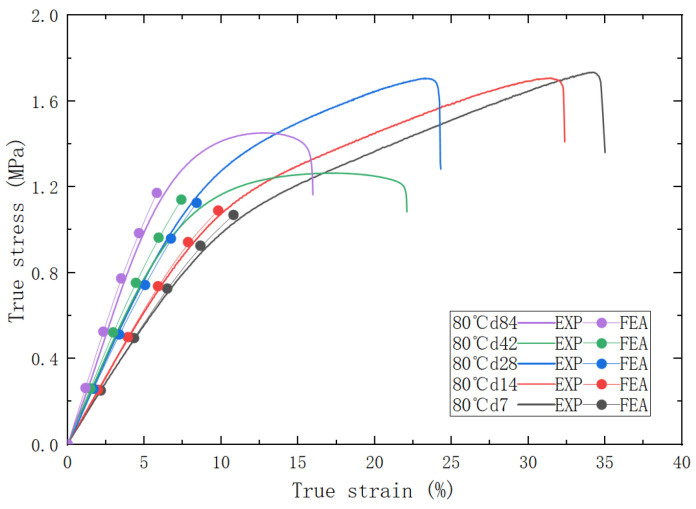
The comparative stress–strain curves of experimental/predicted.

In order to further verify the reliability of the predictive models, the matrix modulus and interface strength at various aging temperatures and aging times were calculated according to the predictive models and used as parameters of the RVE model for simulation. The comparison of stress–strain curves between the simulation and experiments under accelerated aging conditions at 80 °C is shown in Figure 13.

**Figure 14:**
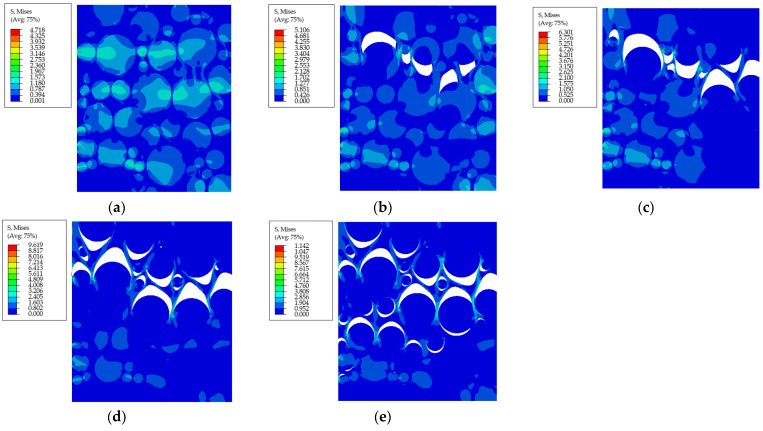
The von Mises stress in RVE with aging properties of 80 °C and (**a**) 7 days, (**b**) 14 days, (**c**) 28 days, (**d**) 42 days, and (**e**) 84 days.

Overall, there is a good agreement between the experimental and predicted curves. With the increase in aging time, it is evident that the modulus of the PFPCs increases, and the dewetting strain decreases. An identical uniaxial tensile strain of 10% is applied on the RVE with various aging properties, as shown in Figure 14.

It can be seen that the matrix modulus increases with the aging time, which leads to greater von Mises stress in the RVE. Under the same load level, the interface between the particles and matrix was well bonded for the RVE, with aging properties of 80 °C for 7 days (Figure 14a). However, for the RVE with aging properties of 80 °C for 14 days, debonding occurred between the large particles and matrix (Figure 14b). With the increase in aging time, the interface strength decreased continuously, and more particles were debonded under the same load level (Figure 14c–e).

**Figure 15:**
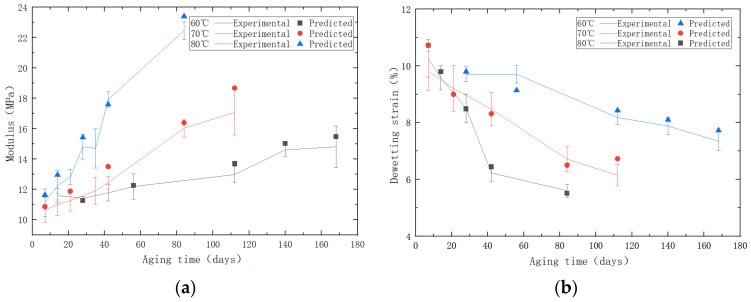
(**a**) The comparative diagram of experimental/predicted modulus. (**b**) The comparative diagram of experimental/predicted dewetting strain.

Through the analysis of the output stress–strain curves, we derived and compared the predicted variations in the modulus and dewetting strain with aging time against the experimental results. The comparison results are shown in Figure 15. The predicted data can be observed to have a similar changing trend as the experimental data. By calculation, the $R^{2}$ values for the modulus and dewetting strain are both 0.94, which verifies the reliability of the predictive models. 

## 5. Conclusions

In this work, experimental and numerical studies were performed to investigate the aging performance of the PFPC material on multiple scales. A novel RVE model, considering the thermo-oxidative aging of the matrix and the degradation of interface strength, was constructed, which can be used to predict the macroscopic aging properties of PFPCs. The detailed conclusions are as follows:

(1) With the increase in aging temperature and time, the modulus of PFPCs increases while the dewetting strain obviously decreases. With the occurrence of an oxidative crosslinking reaction, the crosslink density of the PFPCs increases continuously. In addition, the strength of the interface between the matrix and particles decreases after aging, showing that more particles were debonded on fractured surfaces.
(2) The RVE model of the PFPCs was established using a random particle-packing algorithm. The modulus of an aged matrix in the RVE model was related to its crosslink density according to the theory of polymer physics. The crosslink density was predicted based on the closed-loop chain reaction of polymer oxidation.
(3) The interface between the particle and matrix was modeled by the CZM, and the parameter of cohesive strength in the CZM was determined by inversion techniques. The power function was applied to fit the cohesive strength and crosslink density of the matrix.
(4) By determining the aging effect on the modulus of the matrix and the strength of the interface, respectively, the RVE model was applied to predict the modulus and dewetting strain of the aged PFPCs. The predicted results show good agreement with the test results, which verify the reliability of this novel multiscale modeling framework.

## Figures and Tables

**Table 1:** Table of accelerated aging test conditions.

Temperature (°C)	Aging Time (Days)							
60	14	28	42	56	84	112	140	168
70	7	14	21	35	42	63	84	112
80	7	14	21	28	35	42	63	84

**Table 2:** Table of closed-loop chain reactions.

Reaction Stage		Reaction Equation	Rate Constant
Initiation	I	$2\;\mathrm{POOH}\;\to {{\mathrm{PO}}_{2}}^{*}+P^{*}+H_{2}O+\gamma P=O+(1-\gamma )\;\mathrm{POH}$	k_Ⅰ_
Propagation	II	${P^{*}+O}_{2}\to {{\mathrm{PO}}_{2}}^{*}$	k_Ⅱ_
	III	${{\mathrm{PO}}_{2}}^{*}+\mathrm{PH}\;\to \mathrm{POOH}+P^{*}$	k_Ⅲ_
	IV	$P^{*}+P^{*}\to “\mathrm{inactive}\;\mathrm{products}”$	k_Ⅳ_
Termination	V	$P^{*}+{{\mathrm{PO}}_{2}}^{*}\to \mathrm{POOP}$	k_Ⅴ_
	VI	${{\mathrm{PO}}_{2}}^{*}+{{\mathrm{PO}}_{2}}^{*}\to \mathrm{POOP}+O_{2}$	k_Ⅵ_

where PH represents the polymer substrate; P* is the alkyl radicals; POOH is the hydroperoxide, PO_2_* is the peroxy radical, and POOP is the inactive carbonyl product.

**Table 3:** Statistical table of parameters.

Parameter	Unit	Value
$\nu_{0}$	mol·cm^−3^	$2.66\times 10^{-6}$
$A_{1}$	s^−1^	$1.94\times \;10^{8}$
$A_{2}$	s^−1^	$7.33\times 10^{7}$
$E_{a1}$	J·mol^−1^	$5.424\times 10^{4}\;$[34]
$E_{a2}$	J·mol^−1^	$1.13\times \;10^{5}\;$[34]
${Sol}_{O_{2}0}$	mol·cm^−3^·Pa^−1^	$1.55\times 10^{-12}$ [35]
$H_{sO_{2}}$	J·mol^−1^	$1.2\times 10^{3}\;$ [35]
ω	-	43.64
$\rho_{PH}$	g·cm^−3^	0.913
$M_{PH}$	g·mol^−1^	3$\;\times \;10^{3}$
$V_{PH}/V$	-	0.1594

## Data Availability

Data are contained within the article.
